# Oxidative stress, apoptosis and proliferation in uterus of piglets fed by sow or formula after ex vivo endocrine compound exposure

**DOI:** 10.1038/s41598-025-09895-y

**Published:** 2025-07-28

**Authors:** Malgorzata Wojtaszek, Malgorzata Grzesiak, Olga Pawlikowska, Anna Koziorowska, Marek Koziorowski, Maria Slomczynska, Katarzyna Knapczyk-Stwora

**Affiliations:** 1 Doctoral School of Exact and Natural Sciences, Jagiellonian University, Prof. St. Łojasiewicza 11 Street, Kraków, 30-348 Poland; 2 Department of Endocrinology, Institute of Zoology and Biomedical Research, Faculty of Biology, Jagiellonian University, Gronostajowa 9 Street, Kraków, 30-387 Poland; 3 Department of Biotechnology, Collegium Medicum, Interdisciplinary Center for Preclinical and Clinical Research, University of Rzeszow, Werynia 2, Kolbuszowa, 36-100 Poland; 4 Department of Biotechnology, Collegium Medicum, University of Rzeszow, Pigonia 1 Street, Rzeszow, 35-310 Poland

**Keywords:** Endocrine-active compounds, Uterus, Natural feeding, Artificial feeding, Pig, Reproductive biology, Environmental impact

## Abstract

Endocrine-active compounds (EACs) derived from anthropogenic activities and bioactive components in maternal milk influence neonatal development, a critical period for postnatal uterine morphogenesis. Here, using an ex vivo model, we investigated whether neonatal exposure to the antiandrogen 2-hydroxyflutamide, the environmental estrogen 4-*tert*-octylphenol, and the organochlorine insecticide metabolite HPTE (which exhibits estrogenic, antiestrogenic, and/or antiandrogenic activity) induces oxidative stress and alters proliferation and apoptosis in uterine explants from 10-day-old piglets. Additionally, we assessed whether natural feeding provides protection against the adverse effects of EACs. We found that EACs disrupting androgen or estrogen signaling increased ROS/RNS production, enhanced specific antioxidant enzyme activity, and/or induced apoptosis exclusively in sow-fed piglets, suggesting a compensatory mechanism to maintain cellular homeostasis. Its absence in formula-fed piglets may indicate a reduced capacity to activate protective mechanisms against EACs, potentially due to delayed development. In contrast, EAC-induced alterations in uterine cell proliferation occurred in both feeding groups in a cell type- and feeding-dependent manner. These findings suggest that natural feeding does not fully protect against EAC-induced uterine development disruption, which may have long-term reproductive consequences. Moreover, they reinforce the notion that the neonatal period is a critical window of uterine development, highly sensitive to endocrine disruptors.

**Supplementary Information:**

The online version contains supplementary material available at 10.1038/s41598-025-09895-y.

## Introduction

Over the past several decades, there has been increasing concern regarding the detrimental effects of endocrine-active compounds (EACs), including endocrine-disrupting chemicals, on reproductive health in both animals and humans. These compounds, originating from various anthropogenic sources such as pesticides, industrial chemicals, and pharmaceuticals, have the potential to interfere with the biosynthesis, metabolism, and action of endogenous hormones^1^. Such disruptions can impair endocrine homeostasis, particularly during critical developmental windows, leading to a wide spectrum of reproductive disorders^2^. Uterine development, especially the formation of uterine glands (endometrial adenogenesis), is a pivotal process, as the uterus plays a crucial role in providing the environment for embryo implantation and sustaining pregnancy. It is well established that the uterus is particularly susceptible to the developmental disruptions caused by EACs^3,4^. Epidemiological and animal studies have shown that EAC exposure can adversely affect uterine structure and function, potentially leading to conditions such as endometrial hyperplasia, endometriosis, fibroids and infertility^5,6^.

The postnatal morphogenesis of the uterus is governed by a complex interplay of hormonal, cellular and molecular mechanisms^7^. However, the timing of adenogenesis initiation varies among species. In humans, this process begins prenatally and is completed at puberty^8^. In contrast, in pigs, endometrial adenogenesis initiates on postnatal day 2 and is completed by three months of age^9^. The pig is a valuable model for studying reproductive system development due to the similarity in uterine wall architecture to humans and other domestic animals^10^. During the second week of life, porcine glandular epithelium differentiates from the luminal epithelium, which is characterized by intensive cellular proliferation^11^. In pigs, uterine cell proliferation has been shown to be influenced by estrogens^12^, and neonatal treatment with antiestrogens has been found to alter the biochemical characteristics of the adult endometrium^13^. Furthermore, our studies have demonstrated that *in utero* exposure to the antiandrogen flutamide enhances growth and development of the neonatal porcine uterus, although neonatal flutamide exposure reduces cell proliferation and estrogen receptor alpha expression in immature pig uteri^14^.

Recent studies indicate that some EACs can induce oxidative stress^15^, which significantly contributes to EAC toxicity^16^; however, data on the neonatal uterus are lacking. Oxidative stress results from an imbalance between prooxidants and antioxidants, arising from elevated levels of reactive oxygen species (ROS) and/or reactive nitrogen species (RNS) or reduced antioxidant defense mechanisms, including the activity of catalase (CAT), glutathione reductase (GR), glutathione S-transferase (GST), glutathione peroxidase (GPX) and superoxide dismutase (SOD). EAC-induced oxidative stress is implicated in the pathogenesis of endometriosis and unexplained infertility (reviewed in^17^), as well as in the induction of mitochondrial dysfunction leading to apoptosis^18^.

In addition, bioactive components of the mother’s milk are additional factors influencing development during the neonatal period in both humans and animals^19^. The lactocrine hypothesis of porcine uterine development is well-established^20^. However, the effects of porcine milk on uterine development in piglets exposed to EACs remain poorly understood.

Given these findings, we hypothesize that neonatal exposure to EACs, which modulate androgen and estrogen action, induces oxidative stress and affects cellular proliferation and apoptosis in the developing porcine uterus. We further propose that this effect differs between piglets fed maternal milk and those fed milk replacer. To test this hypothesis, we employed an ex vivo model to examine the solely effects of antiandrogen 2-hydroxyflutamide, the environmental estrogen 4-*tert*-octylphenol, and the organochlorine insecticide methoxychlor metabolite 2,2-bis-(p-hydroxyphenyl)-1,1,1-trichloroethane (HPTE), which possesses mixed steroidal properties (estrogenic, antiestrogenic and/or antiandrogenic), on oxidative stress, apoptosis, and cellular proliferation in uterine explants from 10-day-old sow-fed or formula-fed piglets. Specifically, we measured ROS/RNS levels and antioxidant enzyme activities, assessed proliferating and apoptotic cells using immunohistochemistry and TUNEL, and examined the abundance of proteins implicated in apoptosis and proliferation employing Western blot analysis.

## Results

### Effects of EACs on oxidative stress in the uterus of sow-fed and formula-fed piglets

**Fig. 1:**
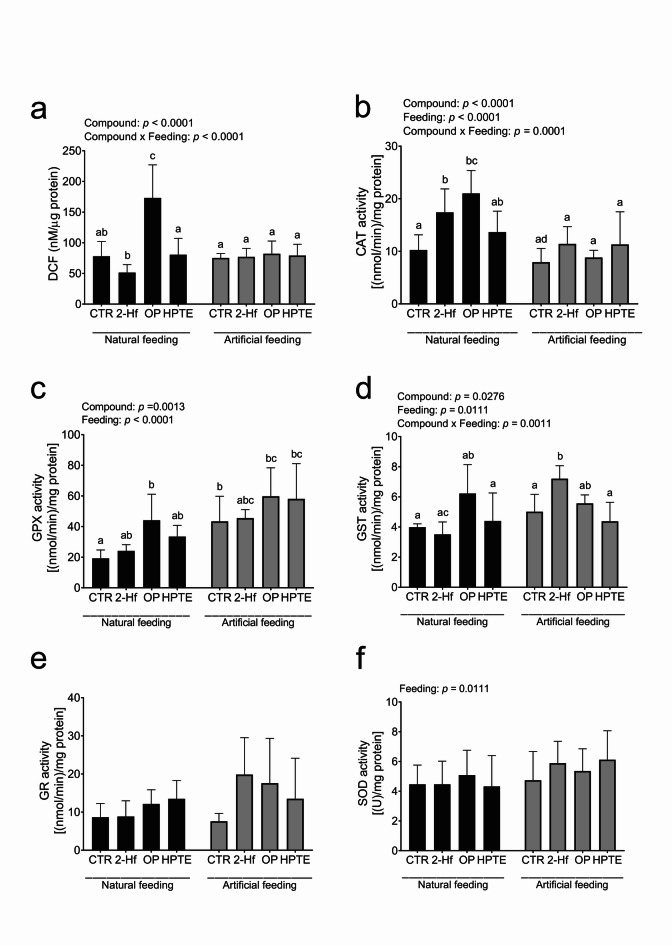
Oxidative stress in uterine explants obtained from control (CTR) and experimental (treated with 2-hydroxyflutamide [2-Hf], 4-*tert*-octylphenol [OP], and 2,2-bis(p-hydroxyphenyl)-1,1,1-trichloroethane [HPTE]) 10-day-old naturally or artificially fed piglets. (a) The total levels of reactive oxygen species and reactive nitrogen species expressed as nM of 2’, 7’-dichlorodihydrofluorescein (DCF) per µg of total protein concentration. (b-f) The activity of antioxidant enzymes: catalase (CAT, b), glutathione peroxidase (GPX, c), glutathione S-transferase (GST, d), glutathione reductase (GR, e), and superoxide dismutase (SOD, f) expressed per mg of total protein concentration. Data are presented as mean ± SD. Data were analyzed using two-way ANOVA with Tukey *post-hoc* test (main effects: compound, feeding), and different letters indicate a significant difference at *p* < 0.05.

To assess the impact of EAC incubation (COMPOUND) and feeding status (FEEDING) on oxidative stress in uterine explants from 10-day-old sow-fed and formula-fed piglets, levels of total ROS/RNS and enzymatic activities of CAT, GPX, GST, GR, and SOD were quantified using fluorimetric or colorimetric assays (Fig. 1).

The total levels of ROS/RNS were significantly influenced by exposure to EACs (*p* < 0.0001) and by the interaction between EAC incubation and feeding status (COMPOUND×FEEDING, *p* < 0.0001), with a 2.2-fold increase (*p* < 0.0001) observed in sow-fed uterine explants incubated with OP compared to sow-fed controls (Fig. 1a). However, the ROS/RNS levels in formula-fed uterine explants remained unaffected by EAC incubation (Fig. 1a).

Both EAC incubation (*p* < 0.0001) and feeding status (*p* < 0.0001), as well as their interaction (*p* = 0.001), significantly affected CAT activity resulting in 1.7-fold and 2-fold increase in sow-fed uterine explants upon incubation with 2-Hf (*p* < 0.01) and OP (*p* < 0.0001), respectively, compared to the sow-fed control group (Fig. 1b). However, CAT activity in formula-fed uterine explants was not affected by EAC incubation (Fig. 1b).

GPX activity was also significantly influenced by both EAC incubation and feeding status (*p* = 0.0013, *p* < 0.0001, respectively). A 2.3-fold increase (*p* < 0.05) in GPX activity was observed in sow-fed uterine explants incubated with OP compared to sow-fed controls (Fig. 1c). Additionally, GPX activity was 2.25-fold higher (*p* < 0.05) in formula-fed control uterine explants compared to sow-fed controls (Fig. 1c). However, EAC incubation did not affect GPX activity in formula-fed uterine explants (Fig. 1c).

In contrast to CAT and GPX activity, the impact of EAC incubation on GST activity was noticed only in the formula-fed group. GST activity was influenced by EAC incubation (*p* = 0.0276) and feeding status (*p* = 0.0111), as well as their interaction (*p* = 0.0011), resulting in 1.4-fold increase (*p* < 0.05) in formula-fed uterine explants upon incubation with 2-Hf compared to formula-fed controls (Fig. 1d).

Incubation with the examined EACs and feeding status did not alter the activity of GR and SOD in uterine explants (Fig. 1e and f).

### Effects of EACs on cell apoptosis in the uterus of sow-fed and formula-fed piglets

**Fig. 2:**
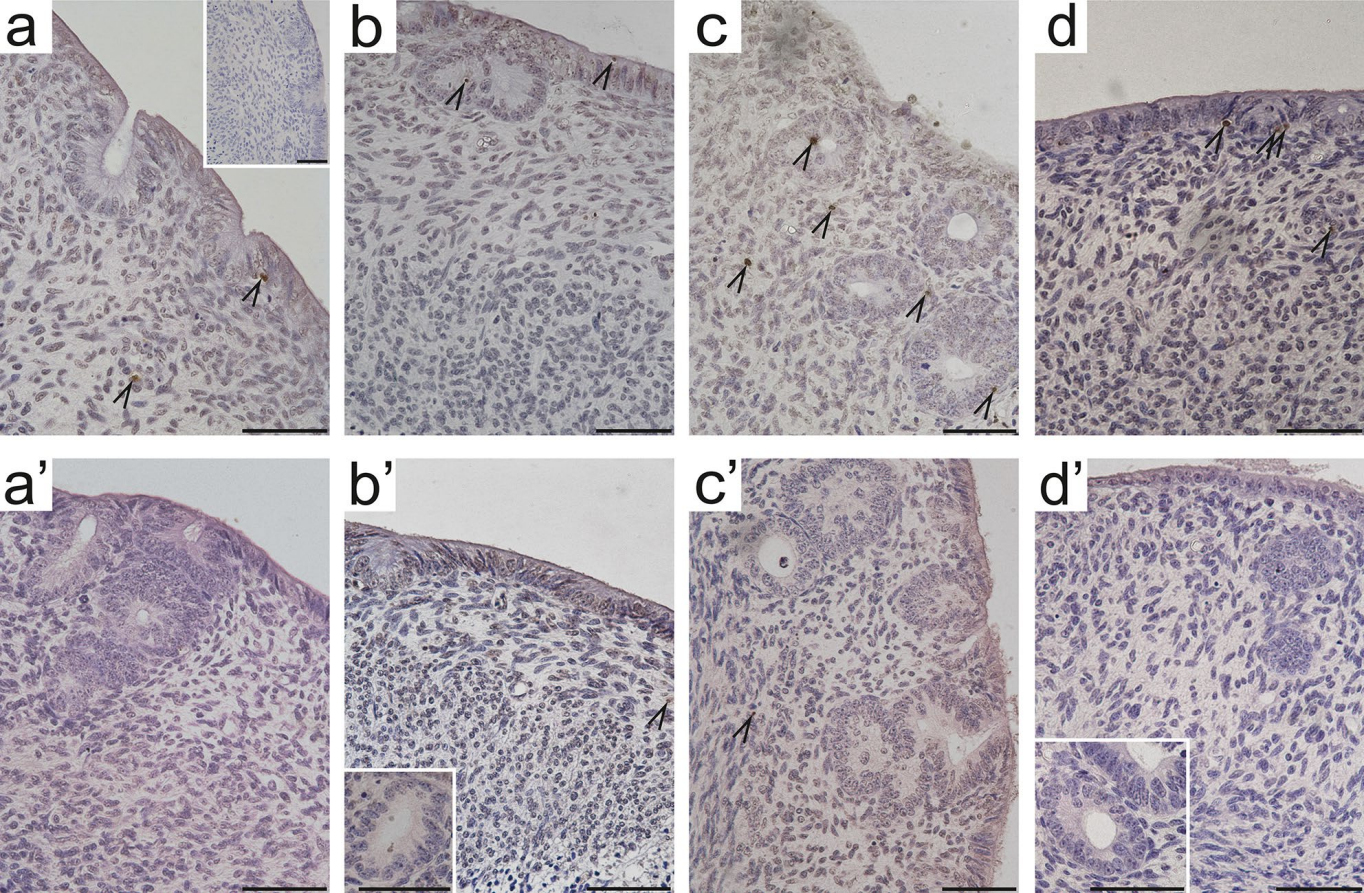
Representative micrographs of in situ localization of apoptotic cells (TUNEL assay) in uterine control explants (a, a’) and explants incubated with 2-hydroxyflutamide (b, b’), 4-*tert*-octylphenol (c, c’), or 2,2-bis(p-hydroxyphenyl)-1,1,1-trichloroethane (d, d’) obtained from 10-day-old sow-fed (a-d) or formula-fed (a’-d’) piglets. Arrowheads indicate single apoptotic cells. Hematoxylin QS was used for counterstaining sections. A negative control was performed without active TdT enzyme (a, inset). All scale bars represent 50 μm.

The number of apoptotic cells in uterine explants from 10-day-old sow-fed and formula-fed piglets incubated with EACs was quantified using TUNEL assay. In situ detection of DNA fragmentation revealed the presence of single apoptotic cells within luminal epithelium (LE), glandular epithelium (GE), and lamina propria (LP) across all examined uterine explants, except for those derived from formula-fed control piglets (Fig. 2). Notably, the effect of EACs on the induction of apoptosis varied depending on the feeding status and type of uterine cells (Table 1). A significant increase in apoptosis was observed exclusively in the sow-fed group following EAC incubation (Table 1, Fig. 2a-d). Specifically, a significant increase (*p* < 0.001) in the number of TUNEL-positive cells within the LE was found upon incubation with 2-Hf compared to controls (Table 1, Fig. 2b). Additionally, the percentage of apoptotic cells in the GE and number of apoptotic cells in the LP were significantly higher (*p* < 0.05 and *p* < 0.01, respectively) following incubation with OP compared to controls (Table 1, Fig. 2c). In contrast, incubation with HPTE did not affect the level of apoptosis (Table 1, Fig. 2d and d’).

**Table 1:** Apoptotic cells in luminal epithelium (LE), glandular epithelium (GE), lamina propria (LP) and myometrium of the uterine explants obtained from control (CTR) and experimental (treated with 2-hydroxyflutamide, 2-Hf; 4-*tert*-octylphenol, OP; and 2,2-bis(p-hydroxyphenyl)-1,1,1-trichloroethane, HPTE) 10-day-old sow-fed (SF) or formula-fed (FF) piglets.

Group	No. of apoptotic cells in LE/100 µm	% of apoptotic cells in GE	No. of apoptotic cells in LP/10,000 µm^2^	No. of apoptotic cells in myometrium/10,000 µm^2^
CTR SF	0.00 (0.00-0.25)^a^	0.00 (0.00–0.00)^a^	0.00 (0.00–1.00)^a^	0.00 (0.00–1.00)
2-Hf SF	5.00 (2.00-6.50)^b^	0.00 (0.00–0.00)^a^	0.00 (0.00–1.00)^a^	0.00 (0.00–0.00)
OP SF	0.50 (0.00–1.00)^a^	0.71 (0.00-2.59)^b^	1.50 (1.00-2.75)^b^	0.00 (0.00–1.00)
HPTE SF	1.00 (0.00-1.25)^a^	0.00 (0.00-2.88)^ab^	1.00 (0.00–1.00)^ab^	0.00 (0.00–1.00)
CTR FF	0.00 (0.00–0.00)^a^	0.00 (0.00–0.00)^a^	0.00 (0.00–0.00)^a^	0.00 (0.00–0.00)
2-Hf FF	0.50 (0.00-1.25)^a^	0.00 (0.00–0.00)^a^	0.00 (0.00–1.00)^a^	0.00 (0.00–0.00)
OP FF	1.00 (0.00–2.00)^ab^	0.00 (0.00–0.00)^ab^	0.00 (0.00–1.00)^a^	0.00 (0.00–0.00)
HPTE FF	0.00 (0.00–0.00)^a^	0.00 (0.00–0.00)^ab^	0.00 (0.00-0.75)^a^	0.00 (0.00–0.00)

^a, b,c, d^ data were analysed using Kruskal-Wallis with Dunn’s *post-hoc* test; median with different superscripts differ significantly (*p* < 0.05); *n* = 5/each experimental group.

The values are expressed as the median ± IRQ (interquartile range).

In addition, Western blot analysis demonstrated the presence of two forms of caspase 3 in all examined uterine explants (Fig. 4a). These included the 35 kDa proenzyme form and the 17 kDa active form of caspase 3. The abundance of caspase 3 was significantly influenced by EAC incubation (*p* = 0.0151) and by the interaction between EAC incubation and feeding status (*p* = 0.0039), with a significant increase (*p* < 0.01) observed in sow-fed uterine explants incubated with 2-Hf compared to sow-fed controls (Fig. 4a). However, the abundance of caspase 3 in formula-fed uterine explants remained unaffected by EAC incubation (Fig. 4a).

### Effects of EACs on cell proliferation in the uterus of sow-fed and formula-fed piglets

**Fig. 3:**
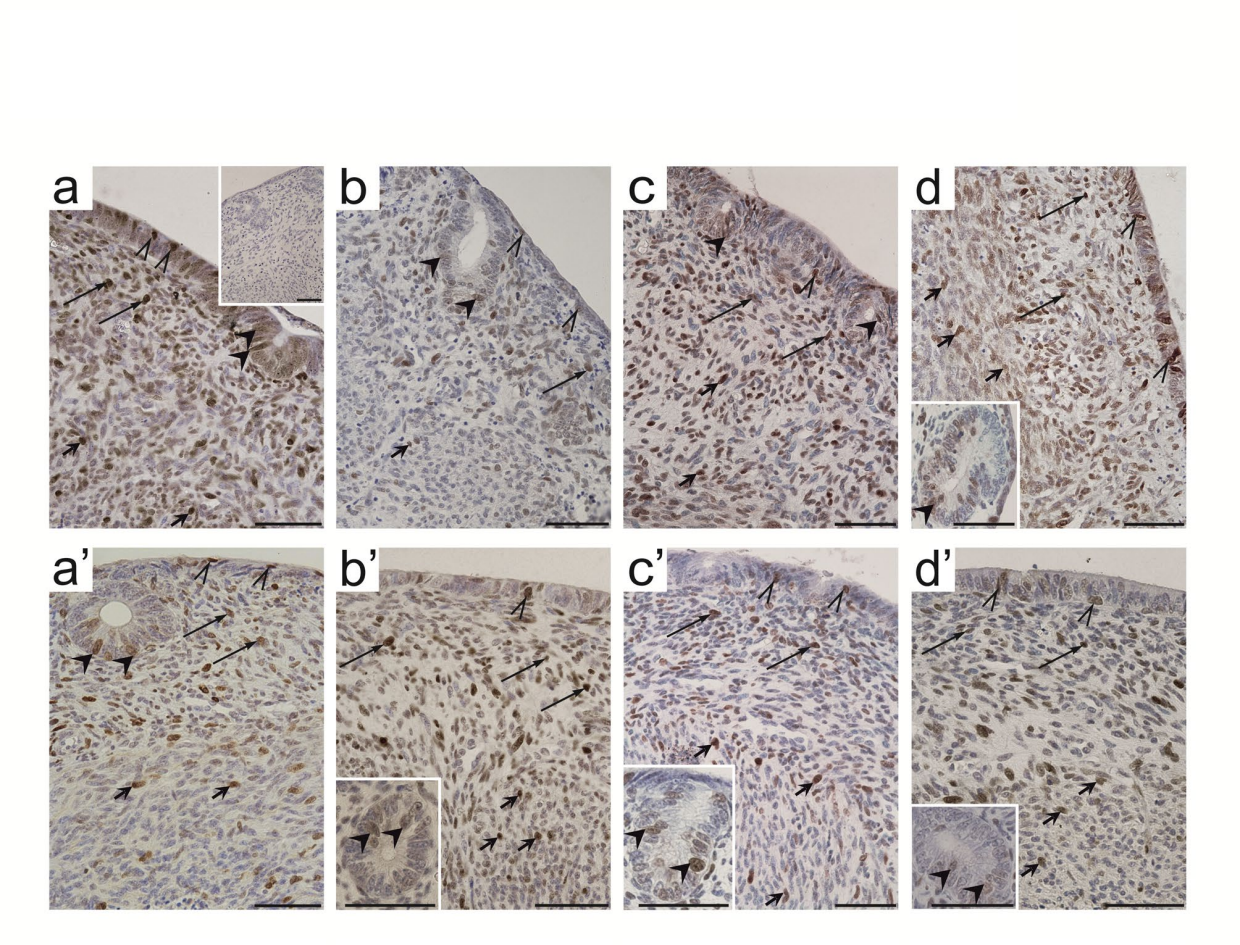
Representative micrographs of the localization of proliferating cell nuclear antigen (PCNA) in uterine control explants (a, a’) and explants incubated with 2-hydroxyflutamide (b, b’), 4-*tert*-octylphenol (c, c’), or 2,2-bis(p-hydroxyphenyl)-1,1,1-trichloroethane (d, d’) obtained from 10-day-old sow-fed (a-d) or formula-fed (a’-d’) piglets. PCNA-positive cells were observed in the luminal epithelium (thin arrowheads), glandular epithelium (thick arrowheads), lamina propria (arrows), and myometrium (short arrows) in all examined groups. Hematoxylin QS was used for counterstaining sections. Negative control sections did not exhibit any positive staining (a, inset). All scale bars represent 50 μm.

The percentage of proliferating cells in uterine explants from 10-day-old sow-fed and formula-fed piglets incubated with EACs was evaluated using immunohistochemical localization of proliferating cell nuclear antigen (PCNA). Positive nuclear PCNA staining was observed in the LE, GE, LP, and myometrium across all examined uterine explants (Fig. 3). Similar to apoptosis results, the effect of EACs on cell proliferation varied depending on feeding status and uterine cell type (Table 2). All results of the two-way ANOVA analysis are provided in Supplementary Table 1 online.

**Table 2:** Percentage of PCNA-positive cells in luminal epithelium (LE), glandular epithelium (GE), lamina propria (LP) and myometrium of the uterine explants obtained from control (CTR) and experimental (treated with 2-hydroxyflutamide, 2-Hf; 4-*tert*-octylphenol, OP; and 2,2-bis(p-hydroxyphenyl)-1,1,1-trichloroethane, HPTE) 10-day-old sow-fed (SF) or formula-fed (FF) piglets.

Group	Percentage of PCNA-positive cells in LE/100 µm	Percentage of PCNA-positive cells in GE	Percentage of PCNA-positive cells in LP/10,000 µm^2^	Percentage of PCNA-positive cells in myometrium /10,000 µm^2^
CTR SF	48.11 ± 8.12^a^	43.18 ± 7.89^a^	50.82 ± 9.73^a^	29.27 ± 7.65^a^
2-Hf SF	39.10 ± 12.83^abc^	35.95 ± 5.18^ab^	37.50 ± 10.40^b^	21.32 ± 6.16^b^
OP SF	36.95 ± 7.31^bc^	39.12 ± 4.43^a^	47.51 ± 6.02^a^	36.84 ± 4.66^ac^
HPTE SF	44.17 ± 10.61^ab^	22.88 ± 8.80^b^	49.19 ± 8.06^a^	41.83 ± 7.73^c^
CTR FF	34.50 ± 12.71^c^	32.60 ± 11.51^ab^	27.24 ± 4.99^c^	8.79 ± 3.35^d^
2-Hf FF	24.29 ± 8.99^d^	43.71 ± 17.07^a^	37.44 ± 12.81^b^	11.53 ± 1.88^e^
OP FF	36.70 ± 9.94^bc^	36.56 ± 7.78^ab^	29.14 ± 8.10^c^	14.98 ± 6.61^e^
HPTE FF	34.27 ± 6.31^bc^	39.62 ± 14.28^a^	29.41 ± 7.89^c^	19.37 ± 4.75^b^

^a, b,c, d,e^ data were analysed using two-way ANOVA with Tukey *post-hoc* test; means with different superscripts differ significantly (*p* < 0.05); *n* = 5/each experimental group.

The values are expressed as the mean ± S.D.

In sow-fed piglets, incubation with the examined compounds generally suppressed uterine cell proliferation, although the effect varied depending on cell type (Table 2). A significant decrease in the percentage of PCNA-positive cells was observed in the LP and myometrium upon incubation with 2-Hf (*p* < 0.001 and *p* < 0.01, respectively) compared to controls (Table 2; Fig. 3b). In addition, incubation with OP significantly decreased (*p* < 0.05) the percentage of PCNA-positive cells in the LE, while incubation with HPTE significantly reduced (*p* < 0.001) the percentage of PCNA-positive cells in the GE (Table 2; Fig. 3c and d). Interestingly, HPTE also significantly increased (*p* < 0.001) the percentage of PCNA-positive cells in myometrium compared to controls (Table 2; Fig. 3d).

In contrast, uterine cell proliferation in formula-fed piglets was elevated following EAC exposure, particularly within the myometrium. The percentage of PCNA-positive cells in myometrium was significantly higher upon incubation with 2-Hf (*p* < 0.01), OP (*p* < 0.001), and HPTE (*p* < 0.001) compared to controls (Table 2, Fig. 3b’-d’). Furthermore, 2-Hf significantly increased (*p* < 0.001) the percentage of PCNA-positive cells in the LP, while reducing (*p* < 0.01) the percentage of PCNA-positive cells in the LE (Table 2; Fig. 2b’).

**Fig. 4:**
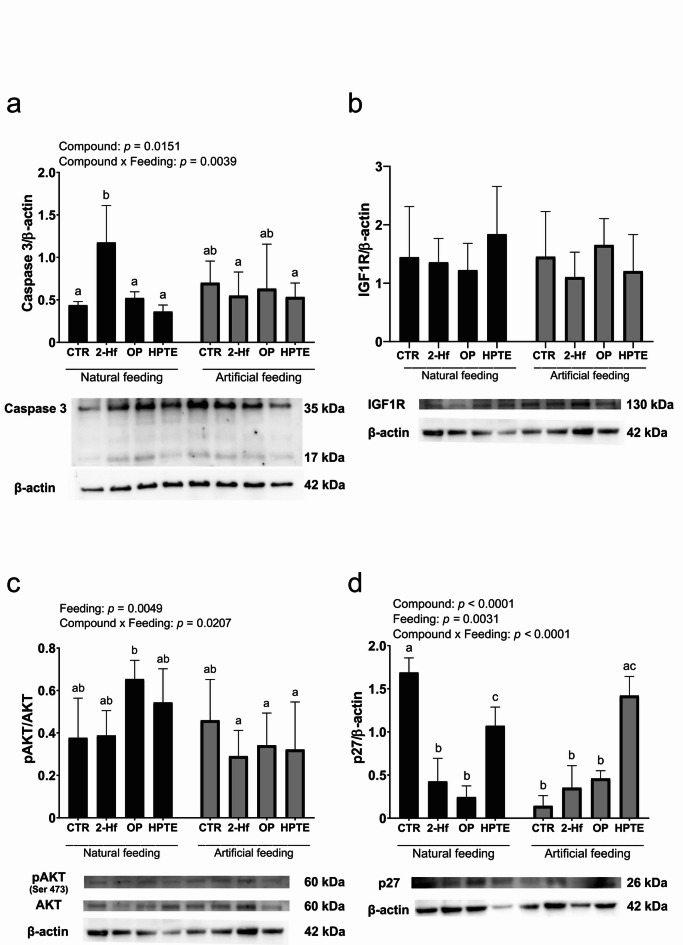
The abundance of caspase 3 (a), IGF1R (b), phospho-AKT (c), and p27 (d) proteins in uterine explants obtained from control (CTR) and experimental (treated with 2-hydroxyflutamide [2-Hf], 4-*tert*-octylphenol [OP]; and 2,2-bis(p-hydroxyphenyl)-1,1,1-trichloroethane [HPTE]) 10-day-old naturally or artificially fed piglets. Relative protein abundance was quantified using densitometry, normalized to β-actin, and representative Western blots are shown (a-d). Uncropped blots are presented in Supplementary Fig. S1 online. Data are presented as mean ± SD. Statistical analysis was performed using two-way ANOVA followed by Tukey’s *post-hoc* test (main effects: compound, feeding), and different letters indicate statistically significant differences (*p* < 0.05).

The abundance of proteins involved in the regulation of cell proliferation was assessed using Western blot analysis, including components of the pro-proliferative insulin-like growth factor 1 receptor (IGF1R)/protein kinase B (AKT) signaling pathway and the anti-proliferative cyclin-dependent kinase inhibitor 1B (p27). The antibodies detected bands at the predicted molecular masses (Fig. 4b-c), and uncropped blots are provided in Supplementary Fig. S1 online. Neither exposure to examined EACs nor feeding status altered IGF1R protein abundance in uterine explants (Fig. 4b). To evaluate IGF1R/AKT pathway activation, AKT phosphorylation at serine 473 (Ser473) was analyzed (Fig. 4c). Phosphorylation of AKT was significantly influenced by feeding status (*p* = 0.0049) and by the interaction between EAC incubation and feeding status (*p* = 0.0207). However, pAKT/AKT levels remained unchanged in both sow-fed and formula-fed uterine explants following EAC incubation (Fig. 4c). In contrast, both EAC incubation (*p* < 0.0001) and feeding status (*p* = 0.0031), as well as their interaction (*p* < 0.0001) significantly affected p27 abundance (Fig. 4d). In sow-fed uterine explants, incubation with 2-Hf (*p* < 0.0001), OP (*p* < 0.0001) and HPTE (*p* < 0.05) resulted in a significant decrease in p27 abundance compared to controls (Fig. 4d). Additionally, p27 abundance was significantly lower in formula-fed control uterine explants compared to sow-fed controls (*p* < 0.0001, Fig. 3b). However, in formula-fed uterine explants, only incubation with HPTE significantly altered p27 levels, leading to a marked increase (*p* < 0.0001) relative to formula-fed controls (Fig. 4d).

In addition, the localization of p27 in uterine explants from 10-day-old sow-fed and formula-fed piglets incubated with EACs was examined using immunohistochemistry (Supplementary Fig. S2 online). Positive p27 staining was predominantly detected in the nuclei of myometrial cells, with additional p27 immunoreactivity in GE and LP across all examined uterine explants (Supplementary Fig. S2 online). Notably, cytoplasmic p27 staining was observed in LE in all examined groups (Supplementary Fig. S2 online).

## Discussion

Our previous in vivo findings indicate that the neonatal period represents a critical developmental widow during which exposure to EACs with androgenic/antiandrogenic or estrogenic/antiestrogenic properties, such as flutamide, OP, and methoxychlor, disrupts early folliculogenesis in 11-day-old piglets^21^ and induces long-term effects on ovarian function observed in adult life^22,23^. To further elucidate the impact of EACs during this sensitive period, we hypothesized that neonatal modulation of androgen and estrogen signaling by EAC exposure induces oxidative stress and alters cellular proliferation and apoptosis in the developing porcine uterus. Moreover, we proposed that these effects vary between piglets fed maternal milk and those receiving milk replacer. This hypothesis was based on our recent findings showing that natural sow-based feeding may provide protection against the effects of EACs on the expression of androgen synthesis-related genes in adrenal glands, highlighting the pivotal role of maternal milk during early neonatal life^24^. Here, using an ex vivo model, we demonstrated that uterine explants from 10-day-old piglets exposed to EACs with antiandrogenic or estrogenic properties exhibit alterations in ROS/RNS levels, antioxidant enzyme activity, and apoptosis primarily in the sow-fed group, except for GST activity, which was affected in the formula-fed group. Furthermore, proliferation analysis indicates that all examined EACs influence uterine cell proliferation in a cell type- and feeding mode-dependent manner. Notably, sow-fed piglets exhibit reduced uterine cell proliferation, whereas formula-fed piglets display predominantly increased myometrial cell proliferation. These findings suggest, for the first time, that maternal milk does not provide protection against EAC-induced adverse effects on postnatal uterine development.

A growing body of evidence indicates that EACs disrupt the tightly regulated redox balance essential for cellular development, growth, and survival, leading to oxidative stress. Dysregulated oxidative status has been implicated in the pathogenesis of various reproductive disorders, pregnancy complications, and infertility (reviewed in^17^). The cellular antioxidant defense system includes enzymatic antioxidants such as CAT, GR, GST, GPX and SOD, which protect cellular biomolecules from oxidative stress by reducing excessive free radical production. In vitro studies using human endometrial stromal cells exposed to the estrogenic compounds bisphenol A (BPA) and p, pʹ-bis-[4-chlorophenyl]-1,1,1-trichloroethane (DDT) revealed a decrease in CAT, SOD and GPX mRNA expression, accompanied by excessive ROS generation^25,26^. Similarly, BPA-induced oxidative imbalance, characterized by decreased antioxidant enzyme expression or activity and increased ROS production, has been reported in rat liver^27^, mouse ovary^28^, porcine oocytes^29,30^, and during the organogenesis of the kidney, brain, and testis in mice^31^. Moreover, maternal BPA exposure disturbed the balance between antioxidants and ROS production, leading to increased oxidative stress in maternal, umbilical cord, and neonatal piglet tissues^32,33^. Additionally, numerous in vivo and in vitro studies have demonstrated that other endocrine disruptors, such as perfluorooctane sulfonate, hexavalent chromium, and phthalates decrease antioxidant enzyme activity, while elevating ROS levels. These mechanisms contribute to endocrine disruption in the testes and ovaries of rats, as well as in mouse ovarian antral follicles^34–36^. The results presented herein demonstrate elevated ROS/RNS levels in sow-fed uterine explants incubated with the estrogenic compound OP, consistently with previous reports on oxidative stress induction by estrogenic EACs. However, unexpectedly, CAT and GPX activities were increased in this group. Given that CAT and GPX are key enzymatic antioxidants involved in ROS detoxification and act as scavengers of hydrogen peroxide^37^, their elevated activity in sow-fed uterine explants exposed to OP may not be a direct effect of OP but rather a compensatory response to increased ROS/RNS levels. This observation suggests a protective mechanism in which antioxidant enzymes are activated in response to OP-induced oxidative stress, underscoring the critical role of CAT and GPX in maintaining uterine redox homeostasis and function. Furthermore, the incubation with antiandrogen 2-Hf resulted in increased CAT activity in the sow-fed group, while GST activity was elevated in the formula-fed group. The observed increase in enzymatic activity suggests that the duration of EAC exposure may have been insufficient to suppress antioxidant enzyme activity, as indicated by the aforementioned literature. Instead, the initial increase in activity of these enzymes may represent an early adaptive response aimed at maintaining redox homeostasis. Moreover, the differential responses between sow-fed and formula-fed uterine explants likely reflect distinct adaptations influenced by early-life feeding status, which may play a regulatory role in modulating antioxidant defense mechanisms.

According to the lactocrine theory, which proposes maternal programming of female reproductive tract development, bioactive components of milk are critical for the trajectory of postnatal porcine uterine development^20^. Lactocrine effects on uterine development in pigs are initiated at the first ingestion of colostrum, with lactocrine signals required to support porcine female reproductive tract development being communicated within 12 h after birth^38^. Disruption of this signaling by milk replacer feeding has been shown to impair endometrial development and negatively impact reproductive performance in adulthood^39,40^. Given that the effects of porcine milk on uterine development in piglets exposed to EACs remain poorly understood, we ensured that newborn piglets received colostrum during the first 12 h after birth and employed ex vivo approach to investigate the potential protective effects of natural feeding against EAC-induced oxidative stress. Interestingly, our findings revealed that EACs with both estrogenic and antiandrogenic activities impacted oxidative stress and/or antioxidant defense mechanisms exclusively in the sow-fed group. In contrast, in the formula-fed group, only GST activity was elevated in response to antiandrogen exposure. These results suggest that while sow-based natural feeding does not confer protection against OP-induced oxidative stress, the antioxidant defense system in the sow-fed uterus may be more responsive to environmental endocrine disruptors. We propose that the development status of the formula-fed uterus may be delayed, potentially limiting its ability to response to EAC exposure compared to the sow-fed uterus. Nevertheless, this notion requires confirmation through further research.

It is well known that ROS play a central role in cell signaling and regulate key apoptotic pathways, as well as other mechanism of regulated cell survival and death, including autophagy and necroptosis^41^. Previous studies have demonstrated that BPA-induced ROS generation triggers apoptosis in porcine oocytes^29,30^. Additionally, environmental estrogens such as zearalenone and nonylphenol have been shown to upregulate proapoptotic factors in the uterus and placenta of sows^42,43^. Our earlier studies on porcine fetal ovaries further revealed that prenatal antiandrogen exposure increased the percentage of apoptotic cells^44^. In the present study, we observed a significant increase in the number of apoptotic cells in uterine explants incubated with antiandrogen (2-Hf) and estrogenic compound (OP), exclusively in the sow-fed group. This finding aligns with our oxidative stress results, supporting a link between redox imbalance and apoptosis. Notably, the apoptotic response varied depending on the uterine cell type, with an increased number of apoptotic cells in the LE following 2-Hf exposure, whereas OP-induced apoptosis was observed in the GE and LP. However, caspase 3 abundance was significantly elevated in sow-fed uterine explants only in response to 2-Hf. We propose that the observed increase in the apoptosis may represent adaptive mechanism aimed at maintaining tissue homeostasis, complementing the increase in antioxidant enzymes activity following OP and 2-Hf exposure. Similar to oxidative stress response, these effects were exclusive to the sow-fed group, suggesting that maternal milk does not provide protection against EAC-induced adverse effects on postnatal uterine development. However, the absence of similar responses in the formula-fed group may indicate a diminished capacity to activate protective mechanisms against EACs, potentially due to delayed developmental status, nevertheless, this hypothesis requires further investigation.

Postnatal uterine development in pigs involves extensive cellular proliferation, leading to the formation of endometrial glands, endometrial folds, and myometrial growth^11^. This process is regulated directly or indirectly by estrogens^12,45^ and is inhibited by neonatal antiestrogen treatment^46^. Our previous study demonstrated that neonatal flutamide exposure reduces uterine cell proliferation and estrogen receptor alpha expression in immature pigs, potentially impairing adult uterine function^14^. In the present study, we assess uterine cell proliferation using PCNA, a well-established proliferation marker^47^. Similar to apoptosis, EAC-induced effects on proliferation were cell-type and feeding-dependent, with the most prominent changes in the myometrium. In sow-fed piglets, EAC exposure generally suppressed proliferation, with 2-Hf significantly reducing PCNA-positive cells in the LP and myometrium, OP in the LE, and HPTE in the GE. However, HPTE increased PCNA-positive cells in the myometrium. In contrast, in formula-fed piglets, EAC exposure stimulated myometrial proliferation, while 2-Hf increased PCNA-positive cells in the LP but decreased them in the LE. In contrast, the abundance of p27 protein, a cyclin-dependent kinase inhibitor that blocks G1 to S phase transitions^48^, was reduced in sow-fed uterine explants exposed to 2-Hf, OP and HPTE, while it was elevated in formula-fed uterine explants incubated with HPTE. These conflicting results may arise from the fact that p27 protein abundance was analyzed in whole tissue, whereas PCNA changes were cell-type specific. Additionally, cytoplasmic p27 staining was observed in the LE across all groups, suggesting a noncanonical role in cell cycle regulation. Notably, p27 function depends on phosphorylation and subcellular localization, with IGF1R/AKT signaling promoting its cytoplasmic sequestration and cell cycle progression^49,50^. However, in the present study, neither EAC exposure nor feeding status altered IGF1R protein abundance and IGF1R/AKT pathway activation. Thus the observed alterations in total p27 levels require additional research to clarify their underlying mechanisms. Overall, our findings indicate that EACs interfering both androgen and estrogen action may disrupt neonatal uterine development by altering cell proliferation in both sow-fed and formula-fed piglets, potentially affecting the trajectory of postnatal uterine maturation. Notably, natural sow-based feeding does not appear to protect against EAC-induced disruption in proliferation during this critical developmental period.

In summary, our results indicate that postnatal exposure to EACs disrupting androgen or estrogen signaling increases ROS/RNS production, enhances certain antioxidant enzyme activity and induces apoptosis exclusively in sow-fed piglets. We propose that this response may serve as a compensatory mechanism to maintain cellular homeostasis, whereas the lack of similar effects in formula-fed piglets may reflect a reduced capacity to activate protective mechanisms against EACs, potentially due to delayed development. This interpretation, however, warrants further investigation. In contrast, EACs affected uterine cell proliferation in both feeding groups in a cell type- and feeding-dependent manner. These findings suggest that the protective effect of sow-based feeding may be tissue-specific and that natural feeding does not provide sufficient protection against EAC-induced disruption in uterine development, which may have long-term reproductive consequences. Moreover, they further support the notion that the neonatal period is a critical window of uterine development, highly sensitive to endocrine disruptors. Nevertheless, it is important to consider the limitations of our study. Long-term in vivo research is needed to fully assess the protective role of maternal milk against EAC-associated effects on uterine development. Additionally, further investigation into the molecular mechanisms underlying EAC action is essential for a more comprehensive understanding of their impact.

## Materials and methods

### Experimental model and sample collection

The use of animals for this research was approved by the Local Ethics Committee at the University of Life Sciences in Lublin, Poland (ethics approval number 42/2023) and all procedures were performed under the supervision of a veterinarian in compliance with the national and EU guidelines for agricultural animal care (EU Directive 2010/63/UE) and the Animal Research: Reporting of in vivo Experiments (ARRIVE) guidelines for the use of animals in research.

Ten newborn female piglets (Large White × Polish Landrace), sourced from different litters, were allowed to suckle their dams for 12 h to get colostrum before being randomly allocated into two groups. The first group (*n* = 5), named the sow-fed group, remained with their respective dams throughout the experimental period and piglets were allowed to breastfeed *ad libitum*. The second group (*n* = 5), assigned as the formula-fed group, received milk replacer (Dolmilk MDP, Dolfos, Poland) *ad libitum*. Uteri were harvested *post mortem* from sow-fed and formula-fed piglets at 10 days of age, and immediately immersed in ice-cold Dulbecco’s Phosphate Buffered Saline (Sigma-Aldrich, St. Louis, MO, USA) supplemented with 1% antibiotic-antimycotic solution (AAS, Sigma-Aldrich). Subsequently, the fresh samples were transported to the laboratory within approximately 2 h of collection. The uterine horns were opened longitudinally, and the tissue was cut into small pieces of approximately 2 mm × 2 mm. These tissue explants were then placed on the top of 1% agarose pillars, with one piece per pillar. The agarose pillars were prepared one day prior to the experimentation as previously described^24^. Uterine explants were pre-incubated for 4 h in Medium 199 without phenol red (M199, Sigma-Aldrich) supplemented with 0.1% bovine serum albumin (BSA, Sigma-Aldrich) and 1% AAS (Sigma-Aldrich), ensuring that the culture medium reached the edge of the pillar without submerging the tissue pieces. Following pre-incubation, the culture medium was replaced with either solely (serving as control) fresh medium (a phenol red-free M199 containing 0.1% BSA and 1% AAS) or supplemented with 2-hydroxyflutamide (2-Hf, 1.7 × 10^− 4^ M), 4-*tert*-octylphenol (OP, 10^− 6^ M), and 2,2-bis-(p-hydroxyphenyl)-1,1,1-trichloroethane (HPTE, 5 × 10^− 7^ M) and uterine explants were incubated for 20 h. The compounds were solubilized in dimethyl sulfoxide (DMSO, Sigma-Aldrich), with the final concentration of the solvent in the culture medium adjusted to 0.1%. The concentration of EACs were chosen based on the literature data^51–53^) and our previous research^24^. All cultures were maintained in 37 °C under a humidified atmosphere comprising 95% air and 5% CO_2_. After incubation period, tissue explants were either snap-frozen in liquid nitrogen or fixed in Bouin’s solution (Sigma-Aldrich), followed by dehydration in an increasing ethanol gradient, clearance in xylene, and embedding in paraplast (Sigma-Aldrich).

### Measurement of the levels of reactive oxygen and nitrogen species

Frozen uterine explants were crushed in liquid nitrogen and mechanically homogenized in ice-cold homogenization buffer (50 mmol/L Tris-HCl, pH 7.6; 150 mmol/L NaCl and 0.1% Triton X‐100) containing 10 µL/mL Halt Protease Inhibitor Cocktail (Pierce Biotechnology, Rockford, IL, USA). The homogenates were centrifuged (10,000 × g at 4 °C for 15 min), and the supernatant was collected and protein concentration was estimated with the method of Bradford^54^. OxiSelect™ In Vitro ROS/RNS Assay Kit (Cell Biolabs, San Diego, CA, USA) was used to evaluate the impact of EACs on the levels of total reactive oxygen and nitrogen species (ROS/RNS) in uterine explants. The assay utilizes the fluorogenic probe dichlorodihydrofluorescin (DCFH) DiOxyQ, which is oxidized to the highly fluorescent 2′, 7′-dichlorodihydrofluorescein (DCF) in the presence of ROS and RNS. Fluorescence intensity, indicative of the total ROS/RNS level, was measured using the Infinite 200 PRO Tecan i-control plate reader at an excitation/emission wavelength of 480/530 nm. All analyses were performed in duplicate. Total ROS/RNS level was expressed as nM of DCF per µg of protein (*n* = 5 per experimental group). Data were expressed as (mean ± S.D).

### Measurement of antioxidant enzyme activities

Frozen uterine explants were crushed in liquid nitrogen, subsequently homogenized on ice in cold Tris/EDTA buffer (50 mmol/L Tris-HCl, pH 7.5 and 1mmol/L EDTA), and then subjected to centrifugation (10,000 × g at 4 °C for 15 min). Enzymatic activities of CAT, GR, GST, GPX, and SOD in supernatants were determined using the following kits (sourced from Cayman Chemical, Ann Arbor, MI, USA): Catalase Assay Kit #707,002, Glutathione Reductase Assay Kit #703,202, Glutathione S-Transferase Assay Kit #703,302, Glutathione Peroxidase Assay Kit #703,102 and Superoxide Dismutase Assay Kit #706,002, according to the manufacturer’s protocols. The CAT activity assay is based on the reaction of enzyme with methanol in the presence of H_2_O_2_ to produce formaldehyde, which is then measured spectrophotometrically at 540 nm using 4-amino-3-hydrazino-5-mercapto-1,2,4-triazole. In the GR assay, enzyme activity was measured by monitoring the rate of NADPH oxidation to NADP+, as indicated by the decrease in absorbance at 340 nm, which is directly proportional to the GR activity. In the GST assay, enzyme activity was assessed by measuring the conjugation of 1-chloro-2,4-dinitrobenzene with reduced glutathione, resulting in an increase in absorbance at 340 nm. The rate of increase is directly proportional to the GST activity in the sample. In the GPX assay, oxidized glutathione, produced upon reduction of hydroperoxide by GPX, is recycled to its reduced state by GR and NADPH. The oxidation of NADPH to NADP + is accompanied by a decrease in absorbance at 340 nm, which is proportional to the GPX activity in the sample. The assay for SOD activity is based on the generation of superoxide radicals through the interaction between hypoxanthine and xanthine oxidase, subsequently detected spectrophotometrically at 450 nm using a tetrazolium salt. All analyses were performed in duplicate using Synergy H1 Microplate Reader (BioTek Instruments, Winooski, VT, USA). The results for CAT, GR, GST and GPX activities were expressed as nmol/min/mg protein, while SOD activity was expressed as U/mg protein (*n* = 5 per experimental group). Data were expressed as (mean ± S.D).

### Western blot analysis

Frozen uterine explants were mechanically homogenized in ice-cold buffer (50 mmol/L Tris-HCl, pH 7.6; 150 mmol/L NaCl and 0.1% Triton X‐100) containing 10 µL/mL Halt Protease Inhibitor Cocktail (Pierce Biotechnology). The homogenates were sonicated and clarified by centrifugation (10,000 × g at 4 °C for 15 min), and the protein content in the supernatants was determined using the Bradford method^54^. Western blots were performed as previously described^21^. Briefly, equal portions of protein (20 µg) extracted from uterine explants (*n* = 4 per each group) were separated on 12% Mini-PROTEAN TGX Precast Protein Gels (Bio-Rad Laboratories Inc., GmbH, Munich, Germany) and transferred to a PVDF membrane (Merck Millipore Ltd., Tullagreen, Carrigtwohill, Co. Cork, IRL). The blotted membranes were blocked for 1 h at room temperature with shaking in Tris-buffered saline (0.05 M Tris-HCl, pH 7.4) containing 0.2% Tween 20 (TBST) with 5% (v/v) non-fat dry milk, followed by overnight incubation at 4 °C with primary antibodies. The primary antibodies included: anti-AKT, anti-phospho-AKT (Ser473), anti-Caspase 3, anti-IGF1R, and anti-p27, which were diluted in TBST buffer (Table 3). Following incubation, secondary rabbit anti-goat (Invitrogen, Rockford, IL, USA), goat anti-mouse (Bio-Rad Laboratories), or goat anti-rabbit (Invitrogen) IgGs linked to horseradish peroxidase were applied (1:3,000; 1 h at room temperature). The chemiluminescent signal was developed using the Westar Hypernova ECL substrate (Cyanagen, Bologna, Italy) and detected using the ChemiDoc XRS^+^ System (Bio-Rad). After washing, each membrane was stripped and reprobed with monoclonal mouse anti-β-actin antibody (Table 3). Relative band density normalized to β-actin was determined using the ImageJ software (National Institutes of Health, Bethesda, MD, USA). Data were expressed as (mean ± S.D).

**Table 3:** List of primary antibodies used.

Antibody	Host species	Clonality	Vendor (catalog no.)	Dilution^a^
Anti-AKT	Rabbit	Polyclonal	Cell Signaling Technology, Beverly, MA, USA; cat. no. 9272	1:1,000 (WB)
Anti-phospho-AKT (Ser473)	Rabbit	Polyclonal	Cell Signaling Technology, Beverly, MA, USA; cat. no. 9271	1:500 (WB)
Anti-Caspase 3	Rabbit	Polyclonal	Cell Signaling Technology, Beverly, MA, USA; cat. no. 9662	1:1,000 (WB)
Anti-IGF1R	Rabbit	Polyclonal	Santa Cruz Biotechnology, Inc., Santa Cruz, CA, USA; cat. no. sc-712	1:100 (WB)
Anti-p27	Goat	Polyclonal	Novus Biologicals, Centennial, USA; cat no. NB100-2816	1:1,000 (WB) 1:100 (IHC)
Anti-PCNA	Mouse	Monoclonal	Millipore, Temecula, CA, USA; cat no. MAB424R	1:200 (IHC)
Anti-β-actin	Mouse	Monoclonal	Sigma Aldrich, Saint Louis, MO, USA; cat no. A2228	1:3,000 (WB)

^a^Application: IHC, immunohistochemistry; WB, Western blotting.

AKT, protein kinase B; IGF1R, insulin like growth factor 1 receptor; PCNA, proliferating cell nuclear antigen; p27, cyclin-dependent kinase inhibitor 1B.

### Evaluation of apoptosis by TUNEL assay

Apoptotic cells in Bouin-fixed, paraffin-embedded uterine explant Sect. (5 µm in thickness) were detected by the terminal deoxynucleotidyl transferase (TdT)-mediated dUTP nick-end labelling (TUNEL) method using the ApopTag Plus Peroxidase In Situ Apoptosis Detection Kit (Chemicon International, Melbourne, Australia) following the manufacturer’s protocol as described previously^44^. Immunodetection was performed using 3,3’-diaminobenzidine (DAB, Sigma-Aldrich) as a chromogen-staining substrate. Sections were counterstained with hematoxylin QS (Vector Laboratories, Burlingame, CA, USA).

The quantification of TUNEL-positive cells (the brown cells) present in three randomly selected sections for each animal was counted from digital images using NIH ImageJ software. The frequencies of apoptotic cells were analyzed within the LE (quantified as the number of TUNEL-positive cells per 100 μm of LE), GE (expressed as the percentage of TUNEL-positive cells within GE), and within the LP and myometrium (expressed as the number of TUNEL-positive cells per at least three random areas of 10,000 µm^2^). Sections were coded and counted blindly. The median value was calculated from 5 animals per experimental group. The data are presented as the median ± IRQ (interquartile range). All pictures were assessed using Nikon Eclipse Ni-U microscope and a Nikon Digital DS-Fi1-U3 camera (Nikon, Tokyo, Japan) with Nikon NIS-Elements Imaging Software BR 4.20.

### Immunohistochemical localization of PCNA and p27

Immunohistochemistry was conducted on Bouin-fixed, paraffin-embedded uterine explant Sect. (5 μm in thickness) as previously described^44^. Prior to incubation with primary antibodies, the blocking step was carried out with either 5% or 10% (v/v) normal horse serum (Sigma-Aldrich) for p27 and PCNA, respectively. Primary antibody against p27 or PCNA was applied overnight at 4 °C. Detailed information on the primary antibodies are given in Table 3. Subsequently, sections were treated with secondary biotinylated horse anti-goat or horse anti-mouse IgG (dilution 1:300; Vector Laboratories) for 1.5 h at room temperature, followed by the incubation with avidin-biotin-peroxidase complex (StreptABComplex-HRP, Vector Laboratories) for 40 min at room temperature. The colour reaction was developed by incubation with DAB. Sections were counterstained with hematoxylin QS. Negative controls were incubated with non-immune mouse or goat IgG instead of primary antibody and processed as described above. Sections were photographed using a Nikon Eclipse Ni-U microscope and a Nikon Digital DS-Fi1-U3 camera with Nikon NIS-Elements Imaging Software BR 4.20.

The quantification of proliferating (PCNA-positive) cells present in three randomly selected sections for each animal was counted from digital images using NIH ImageJ software. The percentage of PCNA-positive cells was evaluated within the LE (quantified as the percentage of PCNA-positive cells per 100 μm of LE), GE (expressed as the percentage of PCNA-positive cells within the GE), and within the LP and myometrium (expressed as the percentage of PCNA-positive cells per at least three random areas of 10,000 µm^2^). Sections were coded and counted blindly. The mean value was calculated from 5 animals per experimental group. The data are presented as the mean ± S.D.

### Statistical analysis

All data were analyzed using GraphPad Prism software v8 (GraphPad Software Inc., La Jolla, CA, USA). Statistical significance was defined as *p* < 0.05. Prior to analysis, normality of distribution for each variable was assessed using the Shapiro-Wilk test. Homogeneity of variance was examined using Levene’s test. Logarithmic transformations were employed as needed to achieve both homogeneity of variance and normality. To evaluate the impact of treatment and feeding status, a two-way analysis of variance (ANOVA) was performed. Significant main effects (*p* < 0.05) were followed by Tukey *post hoc* test. In instances where normality assumptions were not met, the nonparametric Kruskal-Wallis test was utilized to assess differences among groups. *Post hoc* analyses were conducted using Dunn’s test. Specifically, the TUNEL assay results were subjected to nonparametric analysis due to their deviation from normal distribution.

## Electronic supplementary material

Below is the link to the electronic supplementary material.

Supplementary Material 1

Supplementary Material 2

Supplementary Material 3

## Data Availability

The datasets generated during the current study are available in the RODBUK repository at https://doi.org/10.57903/UJ/IXPJF3.
