# Supplementary material for: Oxidative stress, apoptosis and proliferation in uterus of piglets fed by sow or formula after ex vivo endocrine compound exposure
Source: Sci Rep. 2025 Jul 28;15:27386. doi: 10.1038/s41598-025-09895-y (PMC12304335; doi:10.1038/s41598-025-09895-y)
Supplement: Supplementary file 1 — Supplementary Material 1 [file 41598_2025_9895_MOESM1_ESM.docx]

**Supplementary information**

**Oxidative stress, apoptosis and proliferation in uterus of piglets fed by sow or formula after ex vivo endocrine compound exposure**

Malgorzata Wojtaszek, Malgorzata Grzesiak, Olga Pawlikowska, Anna Koziorowska, Marek Koziorowski, Maria Slomczynska, Katarzyna Knapczyk-Stwora

**Supplementary Figures:**

Supplementary Figure S1

Uncropped blots for caspase 3 (a), IGF1R (b), AKT and phospho-AKT (Ser473) (c), and p27 (d) protein abundance in uterine explants obtained from control (CTR) and experimental (treated with 2-hydroxyflutamide [2-Hf], 4-*tert*-octylphenol [OP]; and 2,2-bis(p-hydroxyphenyl)-1,1,1-trichloroethane [HPTE]) 10-day-old naturally or artificially fed piglets. In each lower panel, the corresponding uncropped blot for β-actin protein abundance is showed. Red boxes outline the areas presented in Figure 4. M – marker (PageRuler Prestained Protein Ladder, Thermo Fisher Scientific, Vilnius, Lithuania)

Supplementary Figure S2

Representative micrographs of the localization of p27 in uterine control explants (a, a’) and explants incubated with 2-hydroxyflutamide (b, b’), 4-*tert*-octylphenol (c, c’), or 2,2-bis(p-hydroxyphenyl)-1,1,1-trichloroethane (d, d’) obtained from 10-day-old sow-fed (a-d) or formula-fed (a’-d’) piglets. Staining for p27 was observed in the nuclei of glandular epithelium (thick arrowheads), lamina propria (arrows), and myometrium (short arrows) in all examined groups. Cytoplasmic localization of p27 was observed in the luminal epithelium (thin arrowheads). Hematoxylin QS was used for counterstaining. Negative control sections did not exhibit any positive staining (b’, inset). All scale bars represent 50 µm.

**Supplementary Tables:**

**Supplementary Table S1.** Two-way ANOVA table with compound and feeding as main factors.

|  | **COMPOUND** | | **FEEDING** | | **COMPOUND x FEEDING** | |
| --- | --- | --- | --- | --- | --- | --- |
|  | F | *p* | F | *p* | F | *p* |
| Percentage of PCNA-positive cells in LE/100 µm | 9.50 | <0.0001 | 45.40 | <0.0001 | 5.73 | 0.0009 |
| Percentage of PCNA-positive cells in GE | 2.83 | 0.0464 | 0.735 | 0.3950 | 5.39 | 0.0025 |
| Percentage of PCNA-positive cells in LP/10,000 µm^2^ | 0.258 | 0.0856 | 170 | <0.0001 | 17.00 | <0.0001 |
| Percentage of PCNA-positive cells in myometrium /10,000 µm^2^ | 48.40 | <0.0001 | 446 | <0.0001 | 10.80 | <0.0001 |

Statistical table showing two-way ANOVA results for analyses of PCNA-positive cells in uterine explants with compound and feeding as main factors.
LE, luminal epithelium; GE, glandular epithelium; LP, lamina propria
